# A Doped Polyaniline Modified Electrode Amperometric Biosensor for Gluconic Acid Determination in Grapes

**DOI:** 10.3390/s140611097

**Published:** 2014-06-23

**Authors:** Donatella Albanese, Francesca Malvano, Adriana Sannini, Roberto Pilloton, Marisa Di Matteo

**Affiliations:** 1 Department of Industrial Engineering, University of Salerno, Via Giovanni Paolo II, 132, 84084 Fisciano (SA), Italy; E-Mails: francesca.malvano@gmail.com (F.M.); asannini@unisa.it (A.S.); mdimatteo@unisa.it (M.D.M.); 2 Consiglio Nazionale delle Ricerche, Institute of Atmospheric Pollution Research, Via Salaria Montelibretti, Roma 00015, Italy; E-Mail: pilloton@iia.cnr.it

**Keywords:** gluconic acid, *Botrytis cinerea*, PANI-PAAMPSA, grape, amperometric biosensor

## Abstract

In winemaking gluconic acid is an important marker for quantitative evaluation of grape infection by *Botrytis cinerea*. A screen-printed amperometric bienzymatic sensor for the determination of gluconic acid based on gluconate kinase (GK) and 6-phospho-D-gluconate dehydrogenase (6PGDH) coimmobilized onto polyaniline/poly (2-acrylamido-2-methyl-1-propanesulfonic acid; PANI-PAAMPSA) is reported in this study. The conductive polymer electrodeposed on the working electrode surface allowed the detection of NADH at low potential (0.1 V) with a linear range from 4 × 10^−3^ to 1 mM (R^2^ = 0.99) and a sensitivity of 419.44 nA·mM^−1^. The bienzymatic sensor has been optimized with regard to GK/6PGDH enzymatic unit ratio and ATP/NADP^+^ molar ratio which resulted equal to 0.33 and 1.2, respectively. Under these conditions a sensitivity of 255.2 nA·mM^−1^, a limit of detection of 5 μM and a Relative Standard Deviation (RSD) of 4.2% (n = 5) have been observed. Finally, the biosensor has been applied for gluconic acid measurements in must grape samples and the matrix effect has been taken into consideration. The results have been compared with those obtained on the same samples with a commercial kit based on a spectrophotometric enzyme assay and were in good agreement, showing the capability of the bienzymatic PANI-PAAMPSA biosensor for gluconic acid measurements and thus for the evaluation of *Botrytis cinerea* infection in grapes.

## 1. Introduction

Gluconic acid is one of the most important metabolites associated with grape infection by *Botrytis cinerea* or grey rot*.* This pathogen occurs worldwide, particularly in vineyards exposed to cool and wet conditions during the ripening period. From an oenological point of view the infection of *Botrytis cinerea* must be avoided since it affects negatively the quality of grapes and, therefore, of the resulting wine. Grapes affected by grey rots show a reduction in sugars, acid and polyphenol composition, with problematic alcoholic fermentations, post-fermentation clarity and stability problems [1]. Moreover the wine obtained from *Botrytis cinerea*-infected grapes loses varietal aroma while “dusty”, “dirty” and off flavour appear. Therefore, the presence of gluconic acid is an important marker used by winemakers to estimate the level of *Botrytis cinerea* infection of grapes [2]. The Organization Internationale du Vin (OIV) [3] recommends levels of gluconic acid lower than 200–300 mg·L^−1^, whereas levels up to 1.0 g·L^−1^ indicate an initial stage of fungus infection. Liquid chromatography [4,5], Fourier transform infrared reflectance (FT-IR) spectroscopy [6] and enzyme assays [7–9] have been used for the analysis of gluconic acid. These methods are time consuming and/or require expensive laboratory equipment and trained personnel. The use of biosensors as an analytical method for gluconic acid in grapes, thanks to their high activity, selectivity, ease and rapidity of use, represents an attractive tool for winemakers during the grape purchase step aimed at quickly and easily evaluating their quality.

**Figure 1.**
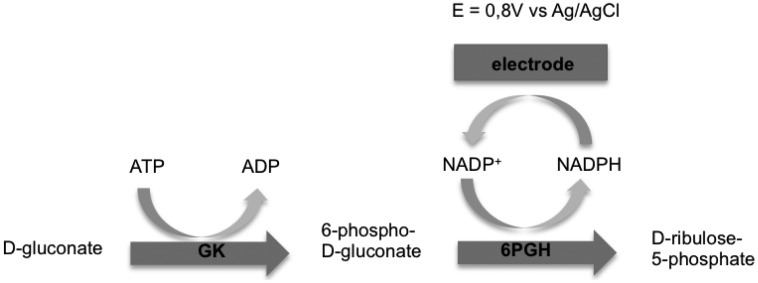
Enzymatic reactions involved for gluconic acid chronoamperometric determination by GK and 6PGDH.

Cetó *et al.* [10] and Del Torno-de Román *et al.* [11] studied amperometric gluconic acid biosensors with a bienzymatic gluconate kinase (GK) and 6-phospho-D-gluconate dehydrogenase (6PGDH) system since gluconate dehydrogenase (GADH) was no longer commercially available. The working principle of these biosensors is represented in Figure 1.

The drawback of this approach is represented by the high overpotential (*ca.* 1 V *vs.* SCE) for the direct electrochemical oxidation of NAD(P)H, which is accompanied by electrode fouling. This overpotential lead to the oxidation of other electroactive species in the sample that interfere with the determination of gluconic acid [12].

To avoid this problem recent approaches have used redox mediators and conducting polymers that let to NAD(P)H oxidation at lower potentials [13–16]. Amongst the various conducting polymers, polyaniline (PANI) [17–19], and its doped forms with poly(2-acrylamido-2-methyl-1-propane sulfonic acid) (PAAMPSA) [14,15], poly(acrylic acid) and poly(styrene sulfonate) have been extensively studied as important conducting materials able to increase the electrical, electrochemical, and optical properties of the sensors.

On the basis of the above discussion this paper reports the development of a screen-printed amperometric biosensor for the determination of gluconic acid based on GK and 6PGDH co-immobilised onto PANI-PAAMPSA polymer. Optimization steps in terms of analytical performance and measurements of gluconic acid content in real samples are reported.

## 2. Experimental Section

### 2.1. Reagents

Aniline (C_6_H_7_N), poly(2-acrylamido-2-methyl-1-propanesulfonic acid, PAAMPSA, M_w_ = kD), poly(ethylene glycol), diglycidyl ether (PEDGE, M_w_ = 500 Da) solution, adenosine 5′-triphosphate disodium salt (ATP) (99%), β-nicotinamide–adenine dinucleotide phosphate (NADP^+^, >90%), β-nicotinamide-adenine dinucleotide phosphate (reduced form, NADPH, >90%), magnesium chloride hexahydrate (99%), D-gluconic acid sodium salt (99%), sodium phosphate dibasic (Na_2_HPO_4_), sodium phosphate monobasic monohydrate (NaH_2_PO_4_-H_2_O) and potassium chloride (KCl) have been purchased from Sigma Aldrich (St. Louis, MO, USA). 6PGDH (EC 1.1.1.44, 150 U/mL) and GK (EC 2.7.1.12, 1500 U/mL) have been purchased from CPC Biotech (CPC Biotech, Napoli, Italy).

### 2.2. Synthesis of PANI—PAAMPSA

Screen-printed carbon electrodes (SPCEs), based on a three electrode (working/auxiliary/reference) layout, were produced in three steps by screen printing different consecutive ink layers on transparent polyester films, as described in Albanese *et al.* [20]. The first layer of a carbon/graphite ink (G-Went, Pontypool, UK) was deposited to define the conducting track and the working electrode, the second one was a silver/silver chloride ink (Acheson Colloiden B.V., city, The Netherlands) used as pseudo-reference electrode, while the third layer consisted in an insulating ink (G-Went).The diameter of the working electrode was 2.8 mm. The electropolymerization of aniline was conducted on the SPCE surface after an electrochemical electrode treatment according to Albanese *et al.* [21]. Conductive PANI-PAAMPSA polymer was electrochemically synthesized by cyclic voltammetry (CV), according to the method described by Bartlett *et al.* [15] with some modifications. SPCEs were soaked in an aqueous solution containing 0.5 M aniline, 1 M hydrochloric acid and 22 g/100 g PAAMPSA; deposition was started by sweeping the potential from −200 to +900 mV on the first scan, to initiate polymer growth; then a potential between −200 and +780 mV, at a scan rate of 50 mV·s^−1^ was cycled 10 times. The resulting film was washed in 1 M HCl solution to remove the reaction products from the film, then five CV scans were performed, from −500 to +500 mV at 50 mV·s^−1^ in 1 M HCl, to fully reduce the film.

### 2.3. Biosensors Manufacturing

6PGDH and GK enzymes were immobilized with PEDGE on PANI-PAAMPSA/SPCE. The immobilization of the enzymes were carried out by dropping consecutive volumes of PEDGE (5 mg·mL^−1^), 6PGDH and GK on PANI-PAAMPSA modified electrode and left overnight to get dried up. The biosensors were stored overnight at 4 °C when not in use.

### 2.4. Measurements

All the electrochemical experiments have been carried out with a PalmSens potentiostat/galvanostat (Utrecht, Netherlands) connected to a personal computer for data recording and visualization. The amperometric measurements have been performed in a Flow Injection Analysis (FIA) apparatus as described in Albanese *et al.* [22]. Measurements have been performed at room temperature.

The biosensors have been placed in a homemade electrochemical wall-jet flow cell while a constant potential of 100 mV *vs.* Ag/AgCl has been applied. Carrier solution PBS with MgCl_2_ 7 mM at pH 7 from reservoir has been pumped with a peristaltic pump (Minipuls 3, Gilson, Villiers Le Bel, France) at 0.5 mL·min^−1^ flow rate to the injection valve (Sample injection valve, Omnifit, Danbury, CT, USA) equipped with a 100 μL sample loop. The use of MgCl_2_, in a concentration between 5 to 7 mM, was necessary because magnesium behaves as an activator of gluconokinase enzyme increasing the current response by a factor between three and six times [23,24], thus 7 mM MgCl_2_ was added to carrier solution. Moreover the bienzymatic scheme required the addition of two cofactors, ATP and NADP^+^, injected in the system with standard (gluconic acid) or real sample solutions so their optimum concentrations have been evaluated.

## 3. Results and Discussion

### 3.1. PANI-PAAMPSA/SPCE

PANI polymer can exist in different forms. The fully oxidized and reduced states, referred to as pernigraniline base (PB) and leucoemeraldine base (LB), respectively, are electrochemically inactive. Emeraldine base (EB) is the intermediate oxidation state of PANI containing an equal number of repeated alternate oxidized and reduced forms (PB and LB). The conductive form of PANI is ‘emeraldine salt’ (ES) and is the protonated form of EB.

PANI polymer used in biosensor applications is the soluble and electrical conductive form of emeraldine which normally exists in acidic media. Acid condition are not favourable for both the activity of enzymes and the stability of NAD(P)H. The use of PAAMPSA during the polymerization of aniline gives to the resulting polymer proper levels of solubility and electrical conductivity at higher pH values in the neutral and alkaline range [14].

**Figure 2.**
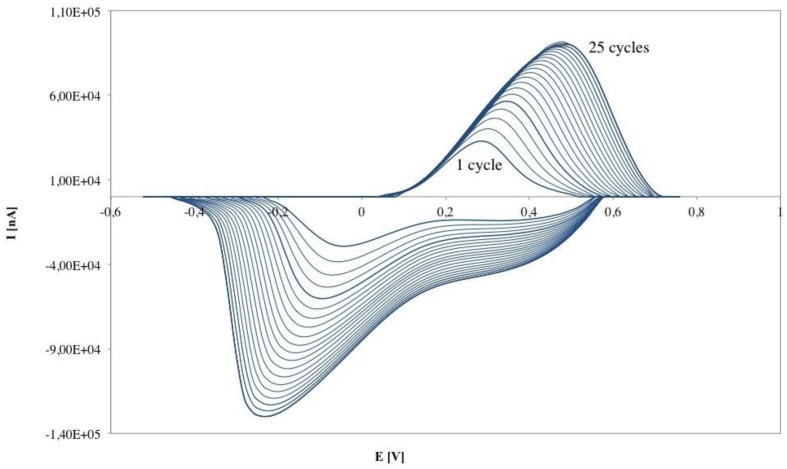
Electrochemical synthesis of PANI-PAAMPSA on SPCE surface.

The electropolymerization of PANI-PAAMPSA film on SPCE, illustrated in Figure 2, shows increasing current during the scans of the CV which indicates the formation and growth of the conducting polymer PANI-PAAMPSA as a thin film on the working electrode. Moreover the increase of current with electropolymerization cycles means that the polymer thickness is increasing [15]. After 20 cycles of CV electropolymerization, the peak currents approached a nearly steady-state value.

**Figure 3.**
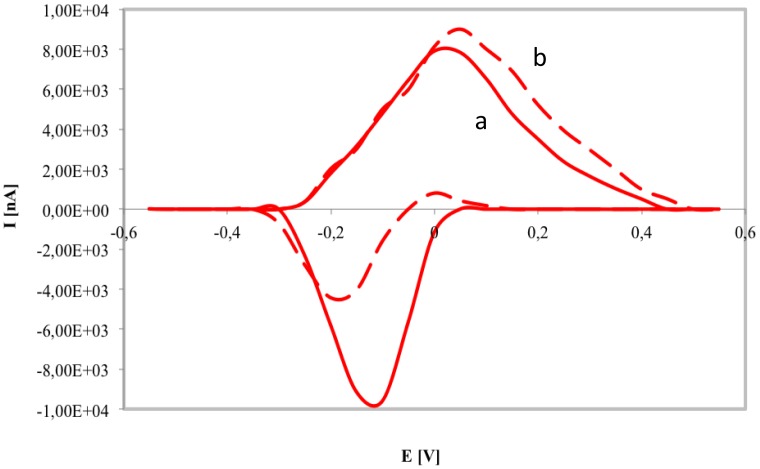
CV of PANI-PAAMPSA/SPCE in PBS, pH 7 without NADPH (**a**) and with 0.4 mM NADPH (**b**).

The capability of PANI-PAAMPSA film as conductive polymer for NADPH oxidation was investigated by CV of PANI-PAAMPSA/SPCE with and without NADPH at 0.4 mM (Figure 3).

During the CV of PANI-PAAMPSA polymer oxidation and reduction peaks were observed between −100 and 50 mV, thus presenting the transitions between the LB/EB and EB/PB states of the PANI [25]. The CV recorded in the presence of NADPH displayed an increase of the anodic peak (at 0.1 V) due to the electrocatalytic oxidation of the NADPH by ES and a significant reduction of the cathodic one. For this reason the working potential at 0.1 V was chosen during the optimization trials of bienzymatic gluconic acid biosensor. Our results were in agreement with Bartlett *et al.* [15] who studied the electrochemical behaviour of PANI film doped by polyvinylsulphonate.

**Figure 4.**
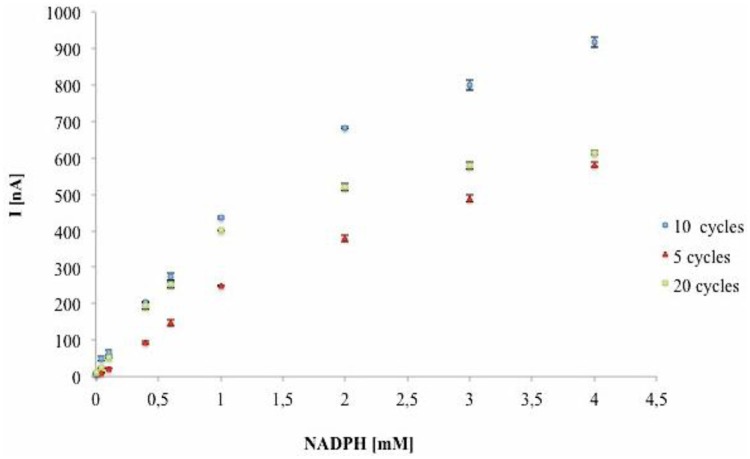
Characterization of NADPH oxidation in PBS pH 7. Calibration curves of PANI-PAAMPSA modified electrodes after electropolymerization with 10 cycles, 20 cycles, 5 cycles.

The effect of polymer thickness on the sensitivity of PANI-PAAMPSA modified electrodes was considered. Different polymer thicknesses were obtained changing the number of CV cycles (5, 10 and 20 scans) during electrochemical polymerization of aniline with PAAMPSA. The sensitivity of different PANI-PAAMPSA modified electrodes was evaluated by chronoamperometry at 0.1 V *vs*. Ag/AgCl injecting NADPH at several concentrations into flow injection system. The results (Figure 4) showed higher currents at same NADPH concentrations for PANI-PAAMPSA/SPCEs prepared with 10 cycles.

The results in Figure 4 showed that a maximum of the current on the WE when PANI is deposited with 10 CV cycles. The current intensity increases between 5 and 10 cycles. The signals measured with the polymer prepared with 20 CV cycles were equal to those obtained at 5 CV cycles, in particular at high NADPH concentrations, highlighting that when the polymeric film is too thick, diffusion phenomena of the analyte in the polymer film or the electron transport to the working electrode are considerably reduced and the sensor sensitivity decreases. These results showed that for a better sensitivity of PANI-PAAMPSA/SPCE towards NADPH oxidation 10 CV cycles are required. For these electrodes a linear range from 4 μM to 1mM (R^2^ = 0.99), a sensitivity of 419.44 nA·mM^−1^ with a Relative Standard Deviation (RSD) of 5.8% (n = 5 PANI-PAAMPSA/SPCEs) have been observed.

### 3.2. Optimization of Bienzymatic Gluconic Acid Biosensor

According to the scheme reported in Figure 1 the optimization of analytical performance of the gluconic acid amperometric biosensor requires the identification of parameters such as GK/6PGDH enzymatic unit ratio, ATP/NADP^+^ molar ratio and best pH conditions.

The optimization of 6PGDH/GK enzymatic unit ratio was performed by immobilizing different amounts of both enzymes on the surface of the modified SPCE. Since the response of the bienzymatic sensor depends on the NADPH generated by 6PGDH, a higher enzymatic unit of this enzyme was used; 0.45 U of 6PGDH represents the maximum amount of the enzyme immobilizable on the electrode surface with the identified immobilization procedure, from an enzymatic solution of 150 U·mL^−1^. The characterization of the biosensors developed was carried out using amount of ATP (7 mM) e NADP^+^ (1.5 mM) and gluconic acid range from 0.01 to 1.5 mM.

As shown in Table 1, higher response in terms of sensitivity and limit of detection have been reached with an enzymatic unit ratio equal to 0.33. The identification of the optimal amounts of cofactors involved in the bienzymatic system was another parameter evaluated for optimizing the gluconic acid biosensor. ATP/NADP^+^ ratio influences the sensitivity of the bienzymatic system but is also important for the cheapness of the measurement due to the high cost of these cofactors.

**Figure 5.**
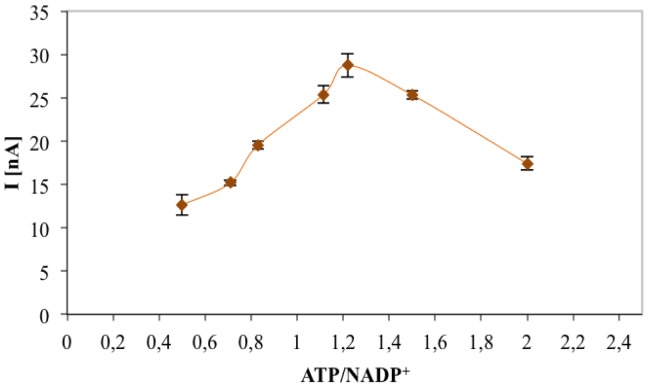
Effect of ATP/NADP^+^ ratio on the amperometric response obtained for 0.1 mM gluconic acid solution.

The optimum ATP and NADP^+^ concentrations were evaluated by registering the maximum chronoamperometric current corresponding to gluconic acid (0.1 mM), measured with an enzymatic unit ratio (6PGDH/GK) of 0.33. Amperometric response for different ATP/NADP^+^ ratios is shown in Figure 5. The current value increased with increasing ATP concentration and reached the highest value with a ATP/NADP^+^ molar ratio of 1.22. The higher amount of ATP needed for the bienzymatic sensor could be justified by the hypothesis that different immobilisation yields for the two enzymes which means an enzyme unit immobilisation ratio in favour of GK with respect to 6PGDH. Further work will involve the optimisation of the immobilisation procedure of the two co-immobilised enzymes: using a different enzyme unit ratio and/or an asymmetrical immobilisation (sequential immobilisation) could reduce the optimal ATP/NADP^+^ ratio to 1.

**Figure 6.**
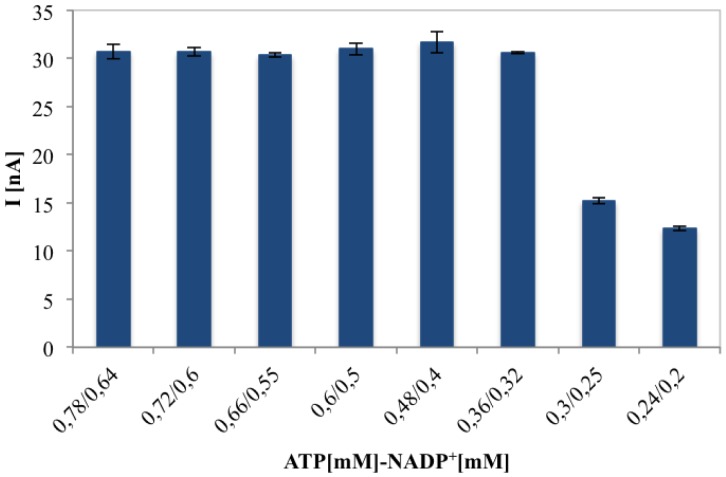
Effect of ATP/NADP^+^ at different concentrations (mM), for their concentration ratio of 1.2 on the amperometric response obtained injecting 0.1 mM gluconic acid solutions into the FIA system.

Figure 6 proves that the biosensor current response for 0.1 mM of gluconic acid solution, remains constant until a certain cofactors concentration, maintaining ATP/NADP^+^ concentration ratio of 1.2 and changing their relative concentrations. Finally 0.36 mM ATP and 0.32 mM NADP^+^ represent the optimised concentrations in terms of sensitivity and cost for each analysis.

According to the results reported above, to obtain the most efficient biosensor performance our devices were prepared utilizing GK 0.15 U and 6PGDH 0.45 U, 0.36 mM ATP and 0.32 mM NADP^+^.

**Figure 7.**
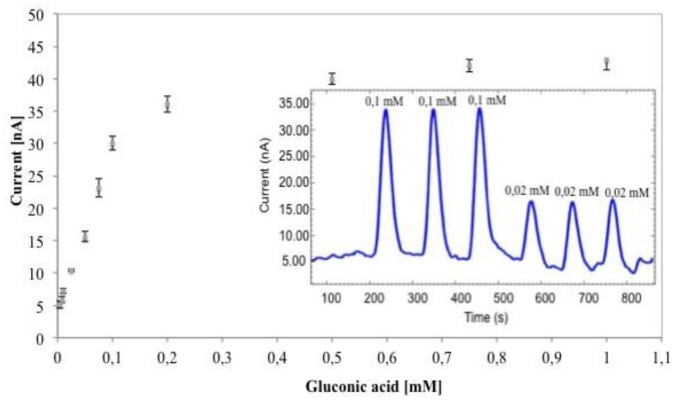
Calibration curve of GK-6PGDH/PEDGE/PANI-PAAMPSA/SPCE biosensor in optimized condition. Insert: amperometric biosensor response obtained injecting gluconic acid solutions into the FIA system.

Finally, the influence of different pH values (ranging from 6 to 8) on the biosensor performance was evaluated with 0.1 mM gluconic acid injections. At pH 7 higher currents were obtained (30 nA *vs.* 23 and 19 nA registered at pH of 6 and 8, respectively), confirming the data previously reported by Tarver *et al.* [14] and Bartlett *et al.* [15] regarding the electrical conductivity of doped PANI film-modified electrodes. Moreover, the optimum pH value measured during our trials was in accordance with del Torno *et al.* [11] and Cetó *et al.* [10] regarding the use of GK and 6PGDH for the development of amperometric biosensors for gluconic acid determination. Calibration curves of the GK-6PGDH/PEDGE/PANI-PAAMPSA/SPCE biosensor under the optimized conditions have been determined by systematic injection of gluconic acid solutions in the concentration range from 0.005 to 0.2 mM (Figure 7). The results are reported in Table 2 together with some previous gluconic biosensors based on bienzymatic systems.

The analytical parameters of the biosensors developed in this study showed similar performance to that reported in the literature except for the sensitivity, that was lower than gluconic acid biosensor reported by Cetó *et al.* [10], who worked at higher potential for the oxidation of NADPH with higher intereferences from real samples and electrode fouling. The reproducibility calculated on five different gluconic acid biosensors at gluconic acid 0.05 mM showed a RSD of 4.2%. Finally, the linear range of the biosensor was 0.005–0.1 mM and its sensitivity was enough to allow the detection of gluconic acid levels established by the OIV which recommends concentrations lower than 200–300 mg·L^−1^.

### 3.3. Repeatability, Operational and Storage Stability

**Figure 8.**
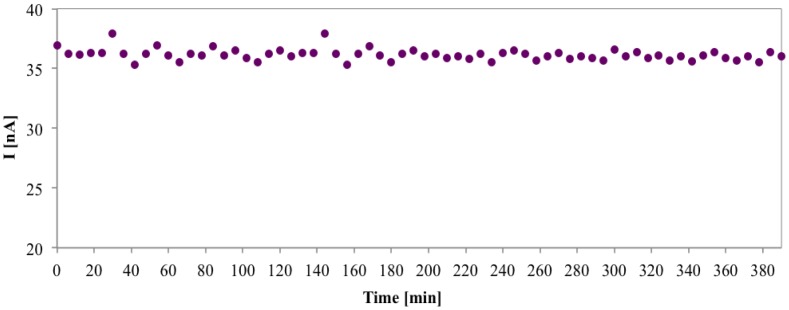
Operational stability of gluconic acid biosensors during 6 h working. Amperometric response to gluconic acid 0.1 mM (70 injections) in PBS, applied potential of 0.1 V *vs.* Ag/AgCl.

The repeatability of the current response to gluconic acid of the same GK-6PGDH/PEDGE/ PANI-PAAMPSA/SPCE was evaluated by consecutive injections of gluconic acid solution (0.1 mM) with a RSD of 2.2% for six injections. Another crucial parameter of the biosensor related to its applicability for the monitoring of food processes is the operational stability, defined as the retention of enzyme activity when it is in use. This parameter was evaluated by injecting 70 samples of gluconic acid solutions (0.1 mM) during an interval of about 6 h. The data reported in Figure 8 shows a high repeatability of the current during the experiment with only 2.6% sensitivity loss during this test period.

Storage stability was also determined to estimate the potential commercialization of the gluconic acid biosensor based on GK-6PGDH/PEDGE/PANI-PAAMPSA/SPCE. For this purpose different gluconic acid biosensors were stored at 4 °C without any chemical preservative (benzoic acid, sodium azide) and characterized at regular interval times. At two months the biosensors preserved their sensitivity but after 90 days a sensitivity decrease of 10% was observed. After this period sensitivity decreased rapidly and no response was recorded after four months of storage.

### 3.4. Analysis of Real Samples

The capability of the proposed biosensor to measure gluconic acid content in grape juice samples was investigated. The matrix effect on the gluconic acid biosensor was then taken into consideration. The study of this effect on the biosensor response is useful to set up the sample preparation in order to minimize the interfering molecules which could be electroactive on the WE electrode surface or act as inhibitors/activators for the enzymes and thus modify the results.

For this purpose the comparison of calibration curves calculated from gluconic acid standard solutions in phosphate buffer and gluconic acid-spiked must samples, from frozen white and red grapes has been carried out. The must samples obtained by pressing red and white frozen grapes were filtered and diluted 10- to 50-fold. The absence of gluconic acid in grapes was first verified, evaluating the absence of anodic peaks after injections of filtered juice into biosensor. In order to quantify the matrix effect the following formula was used:

(1) $$\left[{\text{E}}_{\text{m}}\%=100{\text{S}}_{\text{m}}/{\text{S}}_{\text{b}}\right]$$

where S_m_ and S_b_ represent the sensitivity of biosensors measured with spiked gluconic acid juice samples and gluconic acid standard solutions respectively. The results in Table 3 show that the biosensor sensitivity was affected differently by red or white grape juice. The matrix effect for both types of juice decrease with the increase of dilution factor. Moreover with the same dilution factor, a higher sensitivity decrease was observed for juice from red grapes.

A possible explanation for the different degree of interference of the matrix on the response of the biosensor may be explained by the higher concentration of polyphenols in the red grape skin extracted during sample preparation. The freezing of grape samples caused a skin breakage and thus the leakege of part of the polyphenol content during the crushing of the grapes used for the preparation of the must samples. We conclude that the matrix effect has to be considered for a proper determination of gluconic acid in real samples. Finally, the gluconic acid biosensor reliability during the determination of gluconic acid in different must samples was evaluated by comparison of the results obtained with the biosensor and a commercially available enzymatic kit based on a spectrophotometric assay (Table 4). The results were in total agreement and showed that our biosensor can be very helpful for gluconic acid measurements in must samples and thus for the evaluation of *Botrytis cinerea* infection in grape.

## 4. Conclusions/Outlook

A bienzymatic biosensor to quantify gluconic acid based on the coimmobilization of 6PGDH and GK onto the surface of PANI-PAAMPSA/SPCE has been presented. The use of PANI-PAAMPSA polymers allowed the direct electrochemical oxidation of NADPH at lower potential (0.1 V *vs.* Ag/AgCl) in opposition to the higher potential (0.1 V *vs.* Ag/AgCl) commonly required for carbon electrodes. The linear range, low detection limit, high sensitivity, operational and storage stability, showed the potential of the proposed biosensor as a highly capable analytical device for a fast gluconic acid measurement related to *Botrytis cinerea* in must and grape real samples.

## Figures and Tables

**Table 1.** Calibration parameters of different gluconic acid PANI-PAAMPSA/SPCE biosensors developed by different 6PGDH/GK ratios.

**GK/6PGDH**	**GK [U]**	**6PGDH [U]**	**Sensitivity [nA·mM^−1^]**	**Linear Range [mM]**	**^1^LOD [mM]**	**R^2^**	**^2^RSD (n=3) [%]**
0.33	0.15	0.45	37.49 ± 2.34	0.01–0.1	0.01	0.99	4.58
0.48	0.22	0.45	14.65 ± 2.73	0.05–0.5	0.05	0.99	4.02
1	0.45	0.45	10.66 ± 1.17	0.05–0.5	0.05	0.99	4.61

1 LOD= defined as the gluconic acid concentration that yields a signal-to-noise (S/N) ratio = 3; ^2^ RSD: Relative Standard Deviation calculated for n biosensors.

**Table 2.** Analytical characteristics of bienzymatic GK-6PGDH biosensors for gluconic acid detection.

**Schematic Sensor Assembly**	**E_ap_ [V]**	**Sensitivity [nA·mM^−1^]**	**Linear Range [mM]**	**^1^LOD [mM]**	**Measurement Assembly**	**^2^RSD [%]**	**References**
SPCE/GA-BSA	+0.8	No data	0.007–0.07	0.007	Batch	2.9 (n = 8)	[11]
Epoxy-graphie/PS	+0.8	870	0.007–0.25	No data	Batch	1.7 (n = 3)	[10]
GK-6PGDH/ PEDGE/PANI-PAAMPSA/SPCE	+0.1	255.2	0.005–0.10	0.003	FIA	4.2 (n = 5)	This work

GA: Glutaraldehyde; BSA: Bovine Serum Albumin; PS: Polysulfone membrane; ^1^ LOD= defined as the gluconic acid concentration that yields a signal-to-noise (S/N) ratio = 3; ^2^ RSD: Relative Standard Deviation calculated for n biosensors.

**Table 3.** Matrix effect of grape juice samples on the response of gluconic acid biosensors.

**Sample**	**Dilution Factor**	**Sensitivity nA·mM^−1^**	**Recovery %**
Standard Solutions		274.86 ± 1.34	

Red Grape	1:10	238.03 ± 1.65	86.6

	1:20	264.41 ± 2.45	96.2

	1:30	272.4 ± 1.73	99.1

	1:50	274.74 ± 2.013	100

White Grape	1:10	255.78 ± 1.56	93.06

	1:20	274.31 ± 2.62	99.80

	1:30	274.86 ± 2.71	100

**Table 4.** Results for gluconic acid determination in grapes with the gluconic acid biosensor and a reference enzymatic kit.

**Sample**	*Gluconic Acid mg·L^−1^ Biosensor	*Gluconic Acid mg·L^−1^ Enzymatic Kit
Must A from white grape	15.74 ± 0.45	n.d.^(a)^
Must B from white grape	191.86 ± 1.67	197.62 ± 1.13
Must C from red grape	33.51 ± 1.48	n.d.
Must D from red grape	1187.95 ± 3.56	1127.85 ± 1.24

* All measurements were repeated five times and reported as average ± RSD (n = 5); ^(a)^ The sensitivity of enzymatic kit is in a range between 200–1200 mg·L^−1^.
